# Strain effects on halide perovskite solar cells

**DOI:** 10.1039/d2cs00278g

**Published:** 2022-08-05

**Authors:** Bowen Yang, Dmitry Bogachuk, Jiajia Suo, Lukas Wagner, Hobeom Kim, Jaekeun Lim, Andreas Hinsch, Gerrit Boschloo, Mohammad Khaja Nazeeruddin, Anders Hagfeldt

**Affiliations:** Department of Chemistry – Ångström Laboratory, Uppsala University Box 523 SE-75120 Uppsala Sweden jiajia.suo@kemi.uu.se anders.hagfeldt@uu.se; Laboratory of Photomolecular Science, Institute of Chemical Sciences and Engineering, School of Basic Sciences, École polytechnique fédérale de Lausanne (EPFL) CH-1015 Lausanne Switzerland; Fraunhofer Institute for Solar Energy Systems ISE 79110 Freiburg Germany; Department of Sustainable Systems Engineering (INATECH), Albert-Ludwigs-Universität Freiburg 79110 Freiburg Germany; Institute of Chemical Sciences and Engineering, School of Basic Sciences, École Polytechnique Fédérale de Lausanne (EPFL) CH-1951 Sion Switzerland

## Abstract

Halide perovskite solar cells (PSCs) have achieved power conversion efficiencies (PCEs) approaching 26%, however, the stability issue hinders their commercialization. Due to the soft ionic nature of perovskite materials, the strain effect on perovskite films has been recently recognized as one of the key factors that affects their opto-electronic properties and the device stability. Herein, we summarized the origins of strain, characterization techniques, and implications of strain on both perovskite film and solar cells as well as various strategies to control the strain. Finally, we proposed effective strategies for future strain engineering. We believe this comprehensive review could further facilitate researchers with a deeper understanding of strain effect and enhance the research activity in engineering the strain to further improve performance and especially the device stability toward commercialization.

## Abstract 2

This review systematically describes the origins, characterization and implications of strain in perovskite solar cells and proposes novel control strategies.

## Graphical abstract

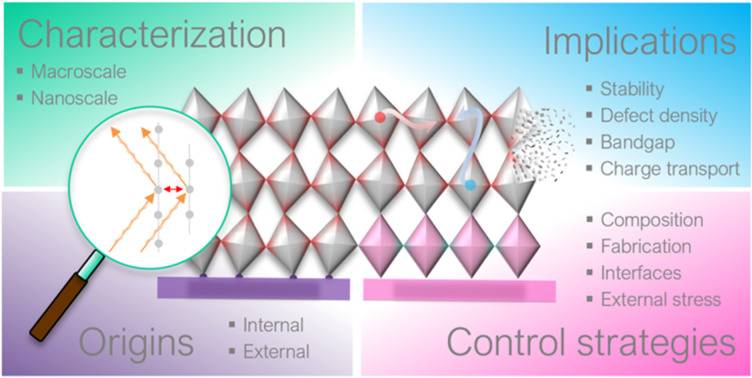

## 1. Introduction

For more than a decade, halide perovskites have been under the spotlight for numerous researchers on photovoltaic applications,^1–3^ owing to their tunable bandgap,^4^ highly efficient light absorption,^5^ high charge carrier mobility,^6^ low recombination rate,^7^ and low-cost solution processing. By optimizing perovskite structure, its nucleation and growth, as well as solar cell constituting layers, the device power conversion efficiency of perovskite solar cells has experienced unprecedented development.^8–10^ However, the mechanically soft nature of perovskites makes them prone to structural strain, which induces significant changes in their properties.^11^

Strain is a well-known physical concept in semiconductors, which can be tailored to modulate their optoelectronic properties. For example, a tunable band structure can be obtained by controlling the strain of WSe_2_ during synthesis *via* utilization of thermal coefficient mismatch between the semiconductor and the substrate.^12^ Incorporating InAs quantum dots (QDs) and GaP tensile strain compensation layers in GaAs solar cells shows enhancement in photovoltaic performance.^13^ In addition, external mechanical stress can control the optical properties of germanium, affecting the band-to-band recombination energy.^14^ Recently, strain engineering has also been found as an important factor in nanocrystalline materials (such as perovskites), the properties of which can significantly deviate from the bulk properties, due to high contribution of surface interactions.^15^ Considering soft ionic nature of polycrystalline perovskite films, it is not surprising to see the rising attention of the scientific community given to strain in such materials, which often exhibit high degree of structural heterogeneity and intrinsic instability. Moreover, strain in halide perovskites was often found to cause additional defects and consequently undesirable non-radiative recombination losses.^16,17^ Simultaneously, several reports mentioned that the slight presence of strain can be beneficial for perovskite device performance.^18^ Therefore, in order to take advantage of the strain in perovskite films and PSCs, a thorough comprehension of its origins and implications is essential for developing effective strategies of strain engineering.

**Fig. 1:**
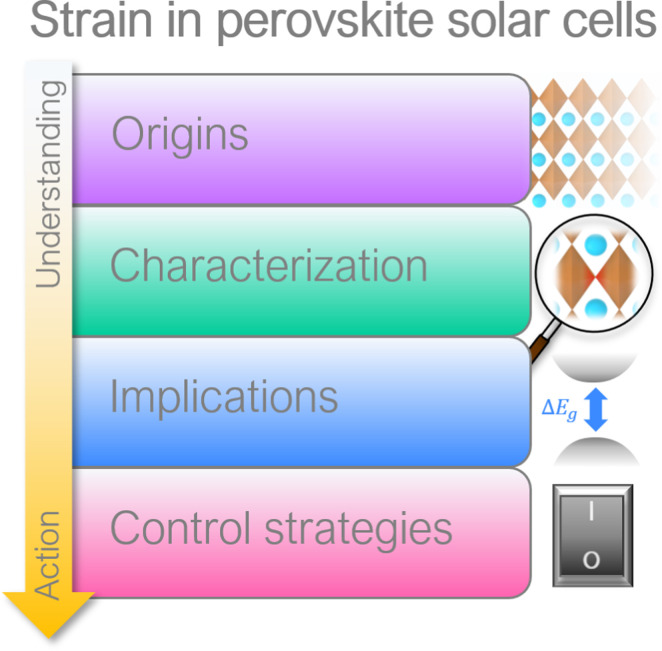
Outline of this review, discussing the origins of strain in halide perovskites, followed by its characterization and implications on opto-eletronic properties, which are finalized by a summary of control strategies for strain engineering.

Currently the exact effect of strain on halide perovskites, particularly in the context of photovoltaic applications, is still under debate and a holistic overview on this topic is missing in the scientific community.^11,18–21^ Therefore, in this timely and comprehensive review, we discuss the causes of strain in halide perovskites with archetypal ABX_3_ structure (where A is a monovalent cation, B is a divalent cation and X is a halide), various methods to characterize it, and its implication on perovskite properties, followed by summary of methods to manage strain in PSCs (Fig. 1). We underline that control of strain is essential for boosting device PCE beyond 26% (with the current certified PCE record being 25.7%^1^) in order to approach radiative limits and bring perovskite closer to its commercialization.

## 2. Strain in perovskite films

### 2.1 Definition

Strain (*ε*) is defined as deformation of crystal structure, caused by applied stress. The strain in a sample is typically quantified by comparing the measured lattice constant(s) with the reference “strain-free” value for the same material:
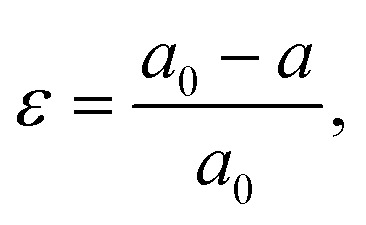
where *a*_0_ and *a* are the lattice constants of strain-free and strained materials, respectively.^22^ The strain is called tensile if the lattice increases in length under applied forces. Conversely, the strain is called compressive if the lattice decreases in length under stress.^23^

### 2.2 Causes of strain in perovskite films

**Fig. 2:**
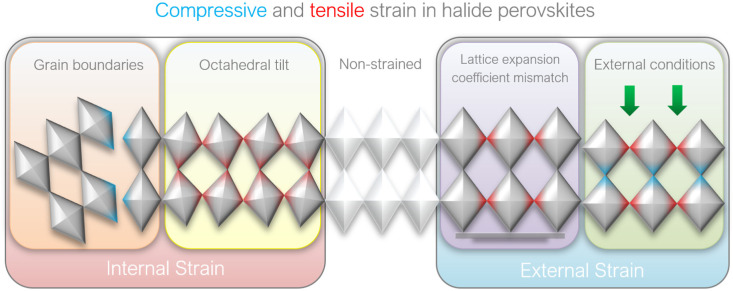
Illustrations of strain origins in halide perovskites.

Fig. 2 illustrates that in perovskite films, strain can be generated (a) internally and (b) externally, depending on the source of stress:

(a) Internal strain is by definition intrinsic in perovskite crystals and is caused by the non-periodicity of crystal lattice in the absence of any external stress. The crystal symmetry disruption in this case typically stems from:

(i) [BX_6_]^4−^ octahedra tilting and change in B–X–B bond angle, thus deviating from the ideal cubic symmetry;

(ii) Heterogeneous crystallization of polycrystalline perovskite films.

(b) External strain is by definition extrinsic in perovskite crystals and is characterized by the distortion of crystal lattice periodicity in the presence of external effects:

(i) Lattice and thermal expansion mismatch between perovskite film and the adjacent substrate during the thermal annealing process;

(ii) External stress conditions (*e.g.* light, temperature, external pressure, applied bias).

#### 2.2.1. [BX_6_]^4−^ octahedra tilting

**Fig. 3:**
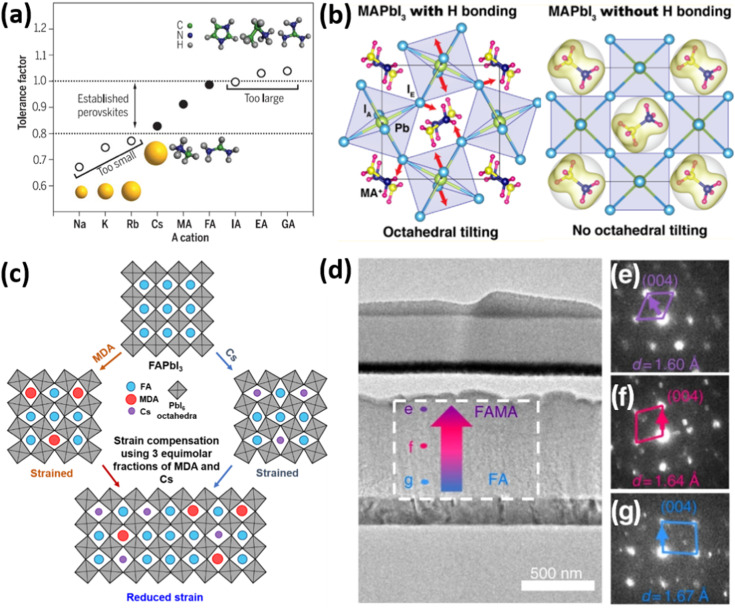
(a) Goldschmidt tolerance factor of APbI_3_ perovskites with different A-cations. Reprinted with permission.^10^ Copyright 2017, The American Association for the Advancement of Science. (b) The effect of hydrogen bonding on the octahedral tilt of MAPbI_3_ perovskites. Reprinted with permission.^24^ Copyright 2016, American Chemical Society. (c) Strain compensation strategies *via* fine-tunement of A-cations. Reprinted with permission.^17^ Copyright 2020, The American Association for the Advancement of Science. (d) TEM image of FIB-polished cross-section of PSC with mixed (FA/MA) perovskite composition, showing (e–g) difference in electron diffraction patterns in [110] zone axis and lattice constant d. Reprinted with permission,^32^ Copyright 2019, Springer Nature.

Deviations of the [BX_6_]^4−^ octahedral orientation in ideal cubic symmetry cause changes in B–X bond length and interaction between their electronic orbitals, resulting in the alternation of electronic band structure of perovskite. As shown in Fig. 3a, among the three cations suitable for incorporation into the cubic APbI_3_ lead-halide perovskite structure, cesium (Cs^+^) has the smallest ionic radius, followed by methylammonium (MA^+^) and formamidinium (FA^+^). If the A-cation's radius is too large or too small relative to the rest of B–X cage, it causes structural distortion tilting the B–X–B bond angle and, thus, creates local lattice strain.^17^ Although these steric effects play the dominant role in the octahedral tilt of inorganic halide perovskites (*e.g.* CsPbX_3_), the hydrogen bonding between organic A-cations and the halides is also considered to be responsible for the octahedral tilt in hybrid halide perovskites such as FAPbX_3_ or MAPbX_3_ (Fig. 3b).^24^*Via* pair distribution function (PDF) analysis, Beecher *et al.* revealed that the typically occurring off-centering and heterogeneous orientation of methylammonium cations (MA^+^) lead to [PbI_6_]^4−^ octahedra tilting, local symmetry-broken state, and continuous change in lattice parameters, resulting in largely heterogeneous strain across the polycrystalline film.^25^ In comparison to other halide perovskites, the cubic α-FAPbI_3_ contains tremendous potential for photovoltaic applications due to favorable thermal stability and energy bandgap. However, its photoactive cubic phase is thermodynamically stable only at temperatures above 150 °C.^26^ Lower temperatures cause a reorientation of FA^+^ cation, favoring the phase transition from cubic to face-sharing hexagonal δ-phases (2H, 4H or 6H).^27,28^ Hence, cation alloying (addition of MA^+^, Cs^+^ or combination thereof) has been often used to obtain more stable FA-rich perovskite films by inducing a slight octahedral tilt, which inhibits the transition from α to δ phase.^29–31^ Such strain induced by the [PbX_6_]^4−^ octahedral tilt could potentially be controlled by fine-tuning the hydrogen bonding degree and balanced partial substitution of A-site cations with suitable cations of different radii (Fig. 3c).^17^ Notably, Zhu *et al.* have demonstrated that alloyed perovskite films such as (FAPbI_3_)_0.85_(MAPbBr_3_)_0.15_, exhibit a correlation between a gradient in elemental composition, namely the higher proportion of smaller MA^+^ cation (relative to FA^+^), and in the in-plane strain confirming that incorporation of A-site cations with different radii, leading to changes in [PbX_6_]^4−^ octahedral tilt, spacing and, consequently, strain (Fig. 3d–g).^32^ However, recent work by Doherty *et al.* demonstrates that although such cation alloying allows to obtain macroscopically homogeneous films, the nanoscopic spatial heterogeneity of A-cations and halides distribution can still result in creation of FA-rich clusters, which seed the impurity phases and ultimately lead to film degradation.^31^

#### 2.2.2. Heterogeneous crystallization

During the nucleation and film growth, slight variations in local environment such as substrate surface morphology, presence of intermediate complexes and concentration gradients result in heterogeneous growth of polycrystalline film.^10,16^ Such crystallization scheme leads to the presence of extended defects such as grain and twin boundaries, where stress is concentrated, and which serve as another source of strain in perovskite.^19^ As was demonstrated by Jariwala *et al.* using electron back-scattered diffraction (EBSD), this strain highly depends on the orientation of grains and interfaces between them.^33^ Recent work by Mela *et al.* revealed substantial Young's modulus (YM) variation across perovskite film with abrupt increase (sometimes by over one order of magnitude) in YM at the grain boundaries.^34^ Moreover, the YM can also vary within the same morphological grain, unveiling the presence of sub-grain domains and even a higher degree of structural inhomogeneity.^34^ Therefore, perovskite films typically exhibit high grain-to-grain orientation spread, local strain heterogeneity, grain boundaries with non-stoichiometric chemical composition and sub-grain lattice orientation disorder.^19,20,33,35^

**Fig. 4:**
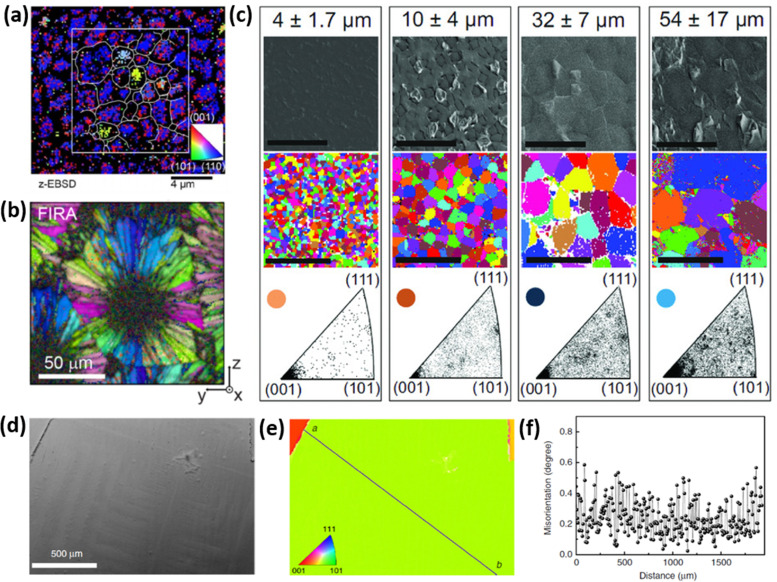
(a) Spatially-resolved EBSD map (in the form of inverse pole figure) showing crystal orientation spread of MAPbI_3_(Cl). Reprinted with permission.^36^ Copyright 2019, Wiley-VCH. (b) EBSD map of MAPbI_3_ perovskite film produced *via* FIRA method, exhibiting spherulitic growth with radially-grown grains. Reprinted with permission.^37^ Copyright 2019, American Chemical Society. (c) SEM top-view image and EBSD maps showing grain orientation spread of MAPbI_3_ films with μm-sized grains (mentioned at the top). The bottom figures indicate the frequency of grain alignment, showing that larger grains, in fact, show higher orientation spread. Reprinted with permission.^38^ Copyright 2018, Wiley-VCH. (d) SEM top-view image of uniaxially-grown MAPbI_3_ film with millimeter-sized grains, produced *via* methylamine liquefaction and recrystallization route. Such films show (e) outstanding grain alignment with (f) negligible misorientation spread. Reprinted with permission,^42^ Copyright 2020, Springer Nature.

However, strain and grain orientation spread induced by perovskite growth process can significantly vary depending on the crystallization kinetics. Fig. 4 presents the inverse pole figures (IPFs) based on the EBSD measurements of perovskite films produced by several different methods, reported in literature. The EBSD map presented in Fig. 4a illustrates the grain-orientation spread in perovskite films manufactured *via* a 2-step crystallization process (mixture of MAI and MACl deposited on top of PbI_2_) with grain sizes reaching a couple of μm size.^36^ Muscarella *et al.* have demonstrated the rapid crystallization process *via* flash infra-red annealing (FIRA), leading to a spherulitic growth mechanism, consisting of >10 μm radially-grown crystallographic domains, as shown in Fig. 4b.^37^ Adhyaksa *et al.* have utilized 1-step deposition of a solution, containing Pb(CH_3_COO)_2_·3H_2_O and MABr dissolved in DMSO to controllably grow films with up to 60 μm (morphological) grain size, by altering the spin-coating speed (Fig. 4c).^38^ All these techniques allowed for the growth of large grains in micrometer-range with significant grain orientation spread, which results in stress at the interface between crystallographic domains. Notably, the larger crystal size, which is typically desired in semiconducting materials due to the expectation of reduced surface carrier trap states, results in higher grain orientation dispersion (bottom IPFs in Fig. 4c), due to the presence of small misoriented crystals trapped in between large grains oriented along [101] and [111] planes.^38^ Regions of high grain orientation spread tend to be more strained, resulting in higher trap densities and lower opto-electronic quality.^33^ Another approach to manufacture perovskite thin films is perovskite liquefaction and recrystallization *via* methylamine gas, which interacts with perovskite precursors differently than conventional solvents like *N*,*N*-dimethylformamide (DMF), dimethyl sulfoxide (DMSO) or *n*-methyl pyrrolidone (NMP).^39–41^ Fan *et al.* developed a method to manufacture films with millimeter-sized grains (Fig. 4d–f), having negligible grain orientation spread and without commonly encountered sub-grain crystallographic domains.^42^ Thus, control over perovskite crystallization kinetics plays an essential role in determining internal strain and can be engineered to obtain favorable opto-electronic properties.

#### 2.2.3. Lattice and thermal expansion coefficient mismatch

Typically, perovskite film is crystallized on a substrate, on which the precursor solution was deposited. Therefore, thermal expansion mismatch between the perovskite films and substrates during thermal annealing process induces strain in the perovskite layer.^43,44^ It has been reported that the tensile stresses in perovskite films introduced by thermal expansion mismatch can reach 50 MPa, sufficient enough to deform some metals like copper.^45^

**Fig. 5:**
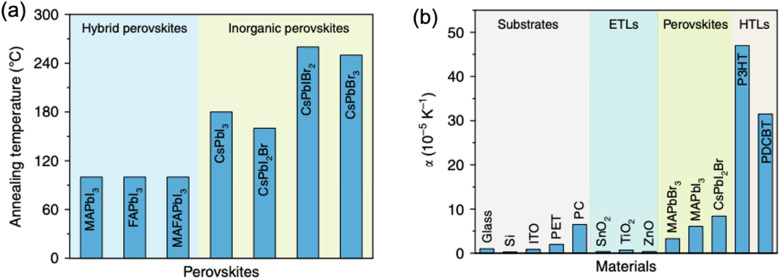
(a) Typical annealing temperatures for hybrid and inorganic perovskites. (b) Lattice expansion coefficients for various commonly employed charge transport layers and halide perovskites in PSCs. Reprinted with permission,^46^ Copyright 2020, Springer Nature.

The correlation between stress (*σ*) and thermal expansion mismatch can be quantified as the following equation:2
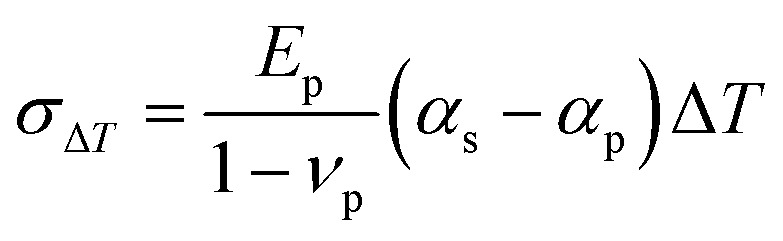
where *E*_p_ represents the perovskite Young's modulus, *ν*_p_ represents the Poisson's ratio of the perovskite, *α*_s_ and *α*_p_ represent the thermal expansion coefficients of the substrate and the perovskite, respectively, and Δ*T* is the temperature gradient of the perovskite film while cooling to room temperature.^45,46^ Moreover, the perovskite films for high-efficiency PSCs require annealing temperatures at least 100 °C in order to guarantee enhanced crystallinity and high-quality films with less defects (Fig. 5a). In particular, comparing to the organic–inorganic hybrid perovskites, the all-inorganic ones need even higher annealing temperatures for conversion of the stable phase structure and crystallization, which makes them suffer from a larger strain.^46^ This indicates that the thermal expansion coefficient (*α*) differences between the perovskite layer and its adjacent layer, along with the high annealing temperature (Δ*T*) are the main sources of induced strain. Thus, such strain effect during perovskite film formation seems unavoidable (with exception of epitaxial growth), because as shown in Fig. 5b, the perovskites present a large difference in thermal expansion coefficients compared to various of the commonly used substrates and their adjacent charge selective layers.

The mismatch in lattice parameters between the semiconducting material and the substrate it is grown on has been particularly well-investigated in the field of III–V semiconductors, which are often used to fabricate multi-junction solar cells. Such mismatch between two adjacent semiconductors induces a residual interfacial strain, which needs to be taken into account during device manufacturing. Hence one of the origins of the external strain in PSCs is the interface between perovskite and an electron- or hole-transport layer it is grown on, which remains even if perovskite would be crystallized at room temperature (hence independent of the temperature coefficient mismatch discussed above).

#### 2.2.4. External stress conditions

**Fig. 6:**
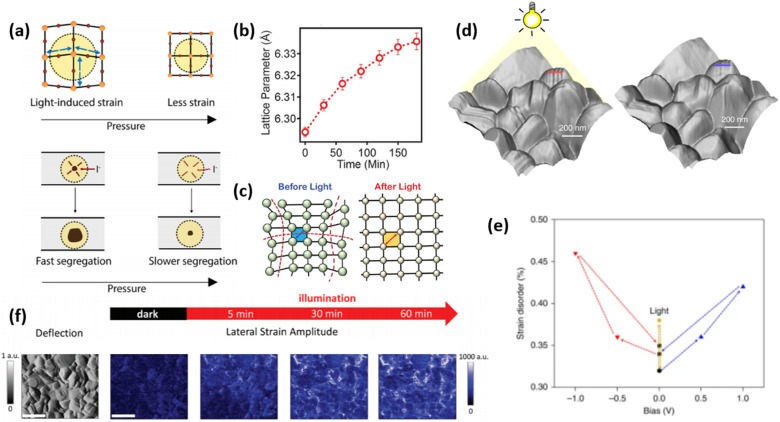
External stress conditions, causing strain in perovskite materials. (a) Illustrations of strain pressure- and light-induced strain, which affects the rate of halide segregation in mixed-halide perovskites. Reprinted with permission.^53^ Copyright 2019, American Chemical Society. (b) Change in lattice parameter of FA_0.7_MA_0.25_Cs_0.05_PbI_3_ perovskite over the illumination duration and (c) proposed mechanism for light-induced lattice expansion. Reprinted with permission.^56^ Copyright 2018, The American Association for the Advancement of Science. (d) Morphological imaging of (FAPbI_3_)_0.85_(MAPbBr_3_)_0.15_ perovskite film surface under light, showing distinct corrugated surfaces, and without light, where the surface is smoother. (e) The effect of applied electrical bias and illumination on strain disorder in such perovskite. Reprinted with permission,^47^ Copyright 2019, Springer Nature. (f) Maps depicting spatial second harmonic PFM response of MAPbI_3_ perovskite films under different illumination duration, allowing to spatially and temporally resolve strain formation (scale bar is 600 nm). Reprinted with permission,^58^ Copyright 2021, Royal Society of Chemistry.

Among external conditions that can induce additional strain in perovskite are illumination, electrical bias, temperature and external pressure – all of which normally occur during the operational lifetime of a PV device.^47,48^ Several strain-related phenomena have been proposed to cause strain in perovskite materials under incident light, such as photostriction^49,50^ (direct conversion of photon energy into mechanical energy and strain), photothermal effect^44,51^ (increase in temperature upon illumination) and build-up of electric field.^52^ Chen *et al.* demonstrated that the thermal expansion upon illumination in MAPbI_3_ perovskites is mainly responsible for the photo-induced strain.^44^ Such thermal expansion of unit cell often leads to reduced activation energy for halide vacancies, resulting in ion migration, weakened Pb–X bond and hence tensile strain (Fig. 6a).^53^ Moreover, Bischak *et al.* proposed that segregated halide-rich regions could originate from the light-induced formation of polarons, towards which the I^−^ ions migrate forming I-rich domains.^54^ Therefore, strain originating from photothermal-induced lattice expansion can accelerate perovskite degradation.^44,55^ On the other hand, however, Tsai *et al.* demonstrated that the opto-electronic properties of the perovskite film can also be positively affected by the photo-induced lattice expansion (Fig. 6b), which the authors attributed to the strain relaxation under light (Fig. 6c).^56^ More recently, Liu *et al.* has decoupled the contribution of light- and thermally-induced contribution to lattice expansion, demonstrating that MAPbI_3_, CsPbIBr_2_ and PEA_2_PbI_4_ perovskites exhibit lattice expansion upon both: external light and temperature stimuli, whereas lattice of FAPbI_3_ only expands upon additional temperature.^57^ Thus, the debate on the role of photo-induced strain on the performance of various perovskite solar cells is still ongoing, highlighting that a more comprehensive understanding of this issue is needed to resolve these ambiguities.

Kim *et al.* have investigated the strain on perovskite surface, influenced by the presence of illumination and/or electrical bias.^48^ The authors found that besides strain disorder induced by illumination, positive electrical bias induces corrugated surface morphology as shown in Fig. 6d and e, while negative bias leads to removal of these uneven features. In contrast, *via* piezoresponse force microscopy (PFM), Qiu and Mativetsky have shown that spatial strain distribution in MAPbI_3_ films changes under electrical bias only in the presence of additional illumination (Fig. 6f).^58^ Strelcov *et al.* have also demonstrated an increase/decrease in piezoresponse under applied negative/positive bias, which the authors attributed to the change in local ion concentration under electric field, which is expected to affect local strain due to migrated ions.^59^

By applying hydrostatic pressure directly to the film or by bending its substrate (in case of flexible foils), bond lengths and angles of perovskite lattice can be modified, inducing compressive or tensile strain.^19,43,60^ Notably, due to structural anisotropy of hybrid perovskite films, the [PbX_6_]^4−^ octahedra deformation is non-uniform under externally applied pressure,^21,61^ leading to highly heterogeneous strain in the film. Typically, perovskite films exhibit a decrease in crystallinity, amorphization and/or phase transformations under high pressure,^62–64^ which can have various effects on perovskite properties, depending on its composition.^21^

Thus, several externally applied strain-inducing conditions can affect the properties of perovskite opto-electronic devices, which might be detrimental or beneficial for the device performance and such external strain needs to be carefully controlled and monitored during the operating conditions.

## 3. Characterization methods for strain on perovskite films

**Fig. 7:**
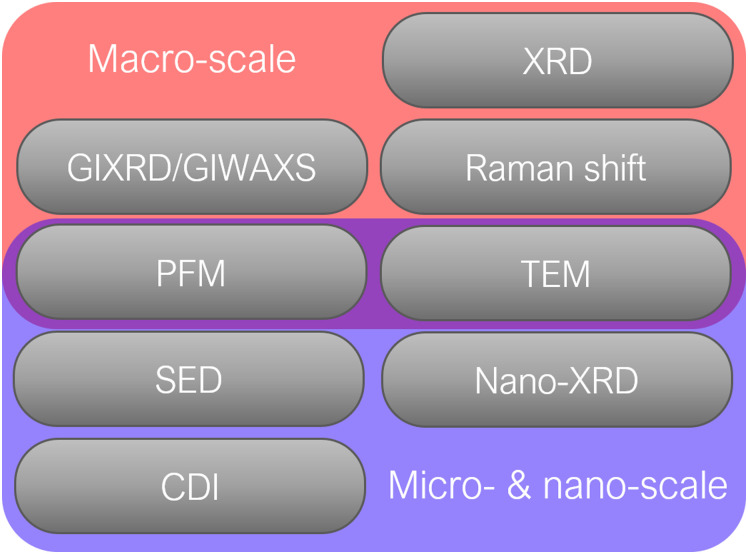
Common characterization methods for analyzing strain in halide perovskites.

Several characterization methods are available to investigate the strain in halide perovskites and its effect on the performance of PSCs. Here, we differentiate between characterization on macro-scale with high spatial averaging and micro- or nano-scale, which allows highly-resolved analysis of strain (as shown in Fig. 7).

### 3.1 Characterization on macro-scale

Since strain induces changes in the lattice *d* spacing of the material, a typical method to detect strain in a sample, is to compare the Bragg peak position obtained from the X-ray diffraction (XRD) pattern with the reference “non-strained” sample. Compressive strain induces the reduction of *d* spacing, while tensile strain causes an increase in *d* spacing, thus, shifting the scattering *q* vector to higher and lower values, respectively. Therefore, strain is commonly expressed in percentages, relative to the non-strained sample, where negative strain values denote compressive strain and positive denote tensile strain. A shift of XRD peak to lower angles (*θ*) indicates a tensile strain, whereas a shift of diffraction peak to higher angles is a signature for compressive strain. However, one of the limitations of the standard XRD measurement in reflection mode is that it characterizes only the out-of-plane strain, and does not account for in-plane strain, which could occur due to Poisson's effect. Furthermore, standard XRD characterization is performed on a relatively large area of a sample, which averages out local instantaneous lattice distortions, thereby, not allowing to resolve strain on a micro- or nano-scale.

**Fig. 8:**
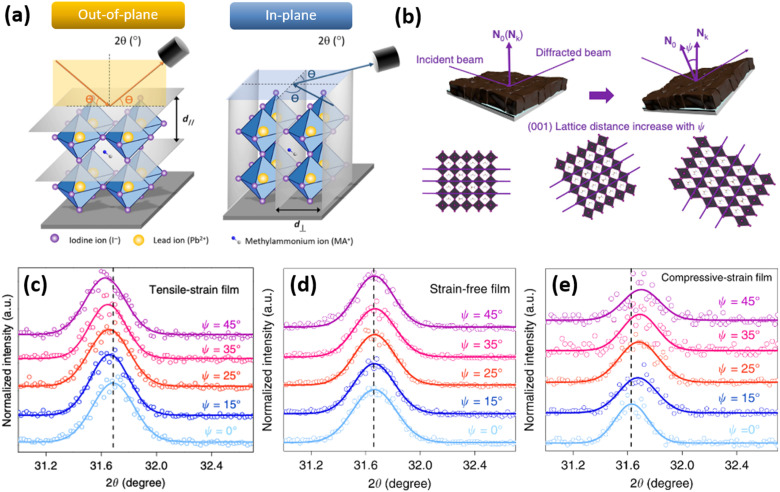
(a) Differentiation between an out-of- and in-plane diffraction-based characterization. Reprinted with permission.^43^ Copyright 2017, The American Association for the Advancement of Science. (b) Illustration of how tilting the instrument angle 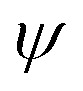 allows to obtain difference between the sample normal vector (*N*_0_) and scattering vector (*N*_*k*_). XRD reflections of the same sample at different grazing angles under (c) tensile strain, (d) no strain and (e) compressive strain. Reprinted with permission,^32^ Copyright 2019, Springer Nature.

To monitor out-of- and in-plane strain (Fig. 8a), grazing incidence X-ray diffraction (GIXRD) measurements can be performed (Fig. 8b). Especially, evaluation on vertical homogeneity as well as the distribution of strain along the perovskite film at a macroscopic level can be quantified by depth-dependent GIXRD through varying the grazing incident angle *ψ* as shown in Fig. 8c–e.^32,43,44^ For example, Zhu and co-workers examined the lattice mismatch induced strain along the mixed halide perovskite film thickness *via* depth-dependent XRD measurements.^32^ It was observed that XRD patterns are similar at different depths (50 nm, 200 nm, and 500 nm), suggesting the same cubic phase structure. However, with increase of the penetrated depths, the diffraction peaks are systematically shifted towards lower angle, indicating the inhomogeneity along the perovskite film with tensile strain (Fig. 8c).

Since Bragg peak broadening can be attributed to variations in *d*-spacing (which are not present in ideal crystal), Bragg peak width analysis can give additional insights into deviations from the average *d* spacing, which characterizes so-called “microstrain”. Williamson–Hall method is commonly employed to quantify the strain based on the different scattering vector dependence on the peak broadening. Assuming that total Bragg peak broadening (*β*_tot_) comes from two contributions: crystal size (*β*_crystal_) and strain (*β*_strain_), their combined influence can be estimated by their convolution:3
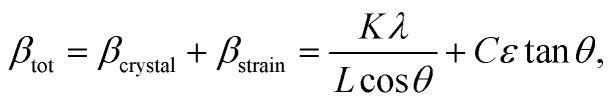
where *K* is a constant, depending on the crystallite shape (usually assumed to be 0.9 or above), *L* is crystallite size, *λ* is incident radiation wavelength and *C* is a constant, which depends on the nature of strain. Multiplying eqn (3) by cos *θ* gives us:4
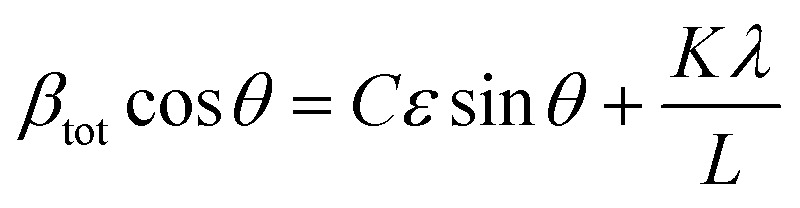
Plotting peak broadening against strain at different diffraction angles gives a linear relationship, called Williamson–Hall plot, in which the strain *ε* and crystal size can be obtained from the slope and the intercept, respectively.

Although, Williamson–Hall analysis is widely used in the perovskite community, one has to note that in order to remove the XRD instrumental peak broadening, a reference perovskite sample must be used, which often substantially differs from the target sample. Furthermore, the assumptions of crystal size, shape and *C* constant are ambiguous, given the versatile nature of hybrid perovskite films, which highlights the importance of keeping these considerations in mind for such analysis.

Raman spectroscopy has also been utilized to measure the residual strain of the perovskite film.^65,66^ The residual strain can generate blue shift of Raman modes, thus, local strain can be evaluated by vibration frequency variations in Raman spectroscopy. For example, through the vibrational modes shift in experimental Raman spectra, Badrooj and co-workers^65^ estimated the amount of compressive strain of MASn_*x*_Pb_1−*x*_I_3_ perovskite film by:5
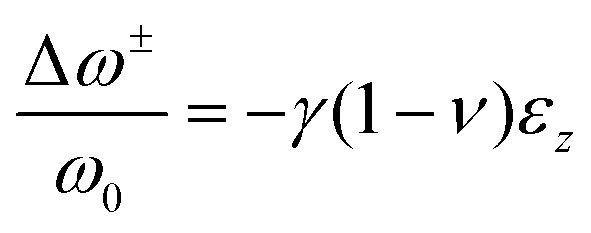
where 
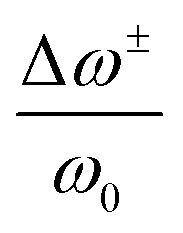
 and *ω*_0_ represent the relative shift of the Raman bands (cm^−1^) and the peak position at zero strain, respectively, *γ* represents the Gruneisen parameter, which can be estimated as 1.6 for polycrystalline perovskites, *ν* and *ε*_*z*_ represent the Poisson ratio (average around 0.3) and the compressive strain, respectively.^65^

Since strain is a common attribute of ferroelastic materials, piezoresponse force microscopy (PFM) can probe the ferroic behavior and identify ferroelastic domains with spontaneous and reversible strain.^67^ PFM is a variation of atomic force microscopy (AFM) measurement during which a sample surface is brought into contact with a conducting probe tip to which an alternating current (AC) is applied. The AC bias induces a piezoelectric effect, causing a deformation of a ferroic surface and deflection of the probing cantilever beam. Several studies have demonstrated the presence of ferroelastic domains in halide perovskite materials, which differ from the morphological grains typically observed form SEM or AFM measurements.^58,68^ Moreover, using PFM, Huang *et al.* clearly demonstrated how strain is created in halide perovskite films upon cubic-to-tetragonal phase transition after perovskite annealing, leading to formation of “stripe-shaped” domains.^68^ In addition, Rothmann *et al.* have utilized a transmission electron microscopy (TEM) under low dosage and rapid acquisition conditions to investigate the creation of strain during phase-transition of CH_3_NH_3_PbI_3_ films.^69^ The authors demonstrated reversible formation of twin domains, which preserve their orientation after undergoing the phase transition, leading to the so-called “twin-memory effect” caused by the presence of crystallization constraints such as strain at the grain boundaries.

Very recently, *in situ* grazing-induced wide-angle X-ray scattering (GIWAXS) technique has been applied in determining the degree of lattice strain and evaluating the relationship between lattice strain and δ-phase suppression of all-inorganic CsPbI_2_Br perovskite during crystal growth. Other techniques, such as photoluminescence (PL), time-resolved PL (TRPL) and transient photocurrent (TPC) can provide complementing evidence of the presence of strain in perovskite film and its effects on opto-electronic properties.^16,32,70,71^

### 3.2 Characterization on micro- and nano-scale

As mentioned earlier, large irradiated area of the sample during a standard X-ray diffraction measurement poses a problem of characterizing the strain on a micro- or nano-scale. In order to reduce the beam footprint and to locally resolve changes in strain, one could utilize an X-ray source with higher brilliance which determines the number of concentrated photons of a certain wavelength on a specific area per unit of time. Thus, brilliance is an important quantification parameter of a light source for microscopy characterization techniques.

**Fig. 9:**
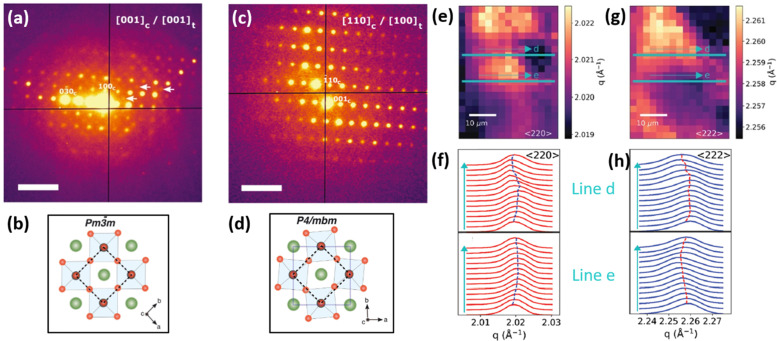
Micro/and nano-characterization techniques of strain. (a) SED patterns of triple/cation perovskite corresponding to [001]_*c*_ zone axis demonstrating the presence of superlattice reflections (white arrows), which are normally forbidden in a cubic *Pm*3*m* structure, often assumed for halide perovskite materials, as shown in (b). Analyzing SED pattern near [110]_*c*_ zone axis in (c) allows to conclude that perovskite has a *P*4/*mbm* structure shown in (d). Scale bar is 0.5 Å^−1^. Reprinted with permission.^31^ Copyright 2021, The American Association for the Advancement of Science. Spatially-resolved nano-XRD measurements using synchrotron radiation, allowing to map variations in scattering vector along specific 〈*hkl*〉 planes and in *d*-spacing. This example demonstrates (e and g) n-XRD map along (e) 〈220〉 and (g) 〈222〉 planes with a change in lattice constant along lines d and e (f and h, repsectively). Reprinted with permission,^16^ Copyright 2021, Royal Society of Chemistry.

One of the diffraction characterization tools, capable of resolving strain on a micro-nano level is scanning electron diffraction (SED) microscopy. Using SED, spatially-resolved diffraction patterns on a nanometer scale can be obtained, which could give insights into local crystallographic properties of the perovskite films (Fig. 9a–d).^15,31^ Consequently, local variations in *d* spacing, grain orientation and strain can be spatially characterized. If coupled with other microscopy and elemental analysis techniques, SED measurements can provide a link between the strain, chemical composition and opto-electronic properties of perovskites.^16,35^

Several recent works utilized synchrotron radiation to perform spatial nano-XRD measurements on perovskite films (example of which is presented in Fig. 9e–h).^16,38,71,72^ Besides a high level of light polarization and collimation, the brilliance of synchrotron radiation is multiple orders of magnitude higher than the standard X-ray tubes, making it the most brilliant source of X-ray photons. This provides immense potential for investigating structural crystallographic inhomogeneities of perovskite films, such as local Bragg peak shifts, localized phase changes or strain variations.^19^

Despite the strong advantages of synchrotron-based nano-XRD measurements, the acquirable information is limited by the incident beam size and by only two dimensions, with an averaged information along the sample depth.^71,73^ Emerging synchrotron-based techniques that help to overcome these limitations is coherent X-ray diffraction imaging (CDI), which does not utilize lenses and is therefore aberration-free with the only resolution limitations being the X-ray wavelength, largest detectable scattering vector and X-ray dose.^15,71,73^ It is a tomography-based method, in which a series of two-dimensional ptychograms are obtained by rotating the sample, which can be reconstructed to produce 3D Bragg diffraction pattern. Applying an inverse Fourier inversion transformation yields a 3D image of the sample with identified crystal domains, unit cell displacement (including all components of the strain tensor) and projection of strain within.^15,19^ Recently, Dzhigaev *et al.* have demonstrated its use in CsPbBr_3_ perovskite nanocrystals, providing in-depth information into the crystallographic twinned domains, lattice tilt and ferroelastic properties. However, the key limitation of this characterization technique for investigating hybrid perovskite materials is their instability under the hard X-rays, which are necessary for this measurement.^73^

## 4. Implications of strain on perovskite films

**Fig. 10:**
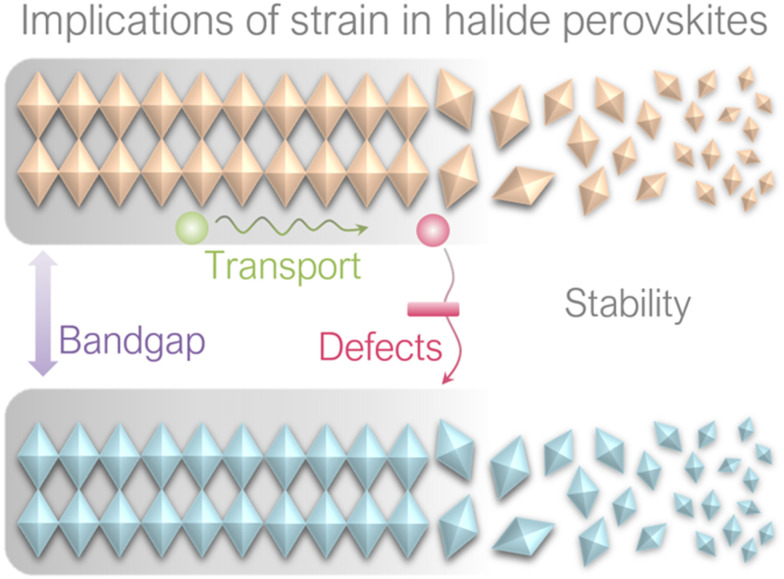
Implications of strain on perovskite films.

It has been reported that strain can induce various effects on perovskite films, including bandgap, carrier transport, defect properties and non-radiative recombination, as well as stability, as illustrated in Fig. 10. These implications will be discussed in this section.

### 4.1 Effect on bandgap

**Fig. 11:**
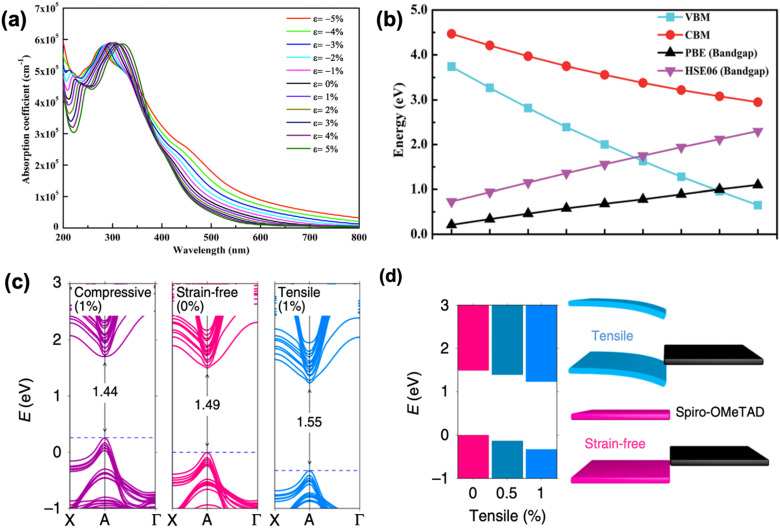
(a) Calculated optical spectra of CsPbI_3_ under different strains. Reprinted with permission.^74^ Copyright 2019, Elsevier. Calculated (b) VBM, CBM and bandgap of CsGeI_3_ as a function of strain. PBE denotes that the DFT calculation was performed using Perdew–Burke–Ernzerhof functional, the accuracy of which could be improved by using a hybrid functional Heyd–Scuseria–Ernzerhof (HSE06) calculations. Reprinted with permission,^75^ Copyright 2019, Royal Society of Chemistry. (c) Calculated band structures of perovskite film under compressive (1%), strain-free (0%) and tensile (1%) strains based on the first-principle DFT approaches. (d) Band-edge energies evolution of perovskite films under gradually increasing tensile strains (left panel), and the schematic of band alignment between hole transporting layer and tensile-strain/strain-free perovskite film (right panel). Reprinted with permission,^32^ Copyright 2019, Springer Nature.

As discussed above, the strain gradient of halide perovskites can be evaluated from the XRD patterns, indicating the crystal structure mismatch along the film, which is mainly attributed to the framework variations (such as shrinkage, enlargement, tilting) of the corner-sharing [PbI_6_]^4−^ octahedra.^32^ As a result, it induces large effects on the optoelectronic properties of a perovskite material and consequently, the device performance. For example, Chen *et al.* investigated the structure of α-FAPbI_3_ under increased compressive strain (from 0% to −2.4%) and revealed its bandgap changes *via* Raman spectroscopy and photoluminescence (PL) spectra, respectively.^66^ A broad and weak peak at around 136 cm^−1^ of the strain-free α-FAPbI_3_ is clearly observed from the Raman spectra, which originated from the lead-iodine bond stretching, while the peak increases in intensity and broadens in width as the compressive strain increases. This suggests a gradual tetragonality of the inorganic framework with increment in the in-plane compressive strain, resulting in a stronger and more distinguishable Raman signal. Further compression of the in-plane and stretching of the out-of-plane lead–iodide bond within the α-FAPbI_3_ structure is evidenced by the broaden peak splitting and shifting under enhanced compressive stress. In addition, the α-FAPbI_3_ undergoes a gradual redshift from 1.523 eV at 0% strain to 1.488 eV at −2.4% strain, corresponding to an estimated 35 meV reduction in bandgap, which was also consistent with their first-principles calculations. Similarly, the optical absorption of inorganic perovskite, CsPbI_3_, also shows a dramatic redshift with accordingly narrower bandgap upon compressive stress, which results in a stronger optical absorption in the visible region (Fig. 11a). In contrast, the absorption edge blueshifts when applying tensile strain on a perovskite material. Calculations show that the bandgap range of CsPbI_3_ can be tuned from 1.03 eV to 2.14 eV by adjusting the strain varying from −5% to 5%.^74^ Moreover, other inorganic perovskite materials, such as CsGeI_3_ and CsSnI_3_ show similar tendency change of bandgap under strain effect (Fig. 11b).^75^

Besides the 3D perovskite films, according to several reports, the bandgap of low-dimensional perovskites can also be modulated through the application of strain. Tu *et al.*^76^ observed that the bandgap of 2D Ruddlesden–Popper (RP) lead iodide hybrid organic-inorganic perovskite film flakes with a general formula of (CH_3_(CH_2_)_3_NH_3_)_2_(CH_3_NH_3_)_*n*−1_Pb_*n*_I_3*n*+1_ responds significantly to uniaxial tensile strain, especially at large *n* (>3). Bandgap increases with the increment of the strain induced, owing to the rotation of the inorganic framework of [PbI_6_]^4−^ octahedra, which consequently stretched the Pb–I bond and lead to an increase in Pb–I–Pb bond length. First-principles calculations also show that the bandgap of 2D MAPbI_3_ presents a linear relationship toward biaxial strain, which increases with tensile strain and decreases with compressive strain. However, the bandgap of 1D MAPbI_3_, on the other hand, exhibits near parabolic response upon strain, which increases under both compressive and tensile strain.^22^

### 4.2 Effect on carrier transport

Studies have demonstrated how residual strain in a PSC affects the hole carrier dynamics and revealed its impact on energy band alignments at the interface between the perovskite and hole-transporting layers.^32,66^ Ultraviolet photoelectron spectroscopy (UPS) illustrates the band structure evolution of α-FAPbI_3_ without strain and with −2.4% strain.^66^ The results show that compressive strain lifts the anti-bonding valence band maximum (VBM) more upward than it does the conduction band minimum (CBM). This is because VBM consists mostly of Pb 6s and I 5p orbitals and interaction between these orbitals becomes stronger under compressive strain, thus pushes the VBM upward.^77^ In contrast, the CBM mainly consists of nonbonding localized states of Pb 6p orbitals, which is less sensitive to the [PbI_6_]^4−^ octahedral deformation.^78^ As a result, the better energy alignment between the VBM of the perovskite and the Fermi level of the Au contact combined with a higher carrier mobility under compressive strain provides an enhancement in photocurrent of the PSCs. Similarly, through first-principles calculations, Zhu *et al.* found that the bandgap decreases as the perovskite film experiences compressive strain compared to a strain-free sample, whereas the bandgap increases under exposure to tensile strain, as shown in Fig. 11c.^32^ Moreover, they unraveled dual effects of the VB downward bending as shown in Fig. 11d: (1) it induces an electric field at the interface that acts as a barrier for efficient hole extraction and (2) also cause deeper level defects in the perovskite film. In contrast, the opposite VB shift of the perovskite under compressive strain provides a favorable energy alignment at the perovskite/HTL interface, resulting in a preferable charge transfer.

In addition, it was shown that strain could also influence the conductivity of the perovskite bulk material. Evidenced by DFT calculations combined with the Green's functional formalism, Berdiyor and co-workers found a decrease in conductivity of the perovskite material under tensile strain, owing to the localization of charge carriers; however, an increase in charge transport of the perovskite material under compressive strain, because of the enhanced overlap of the atomic orbitals.^79^

### 4.3 Effect on defect properties and non-radiative recombination

Metal halide perovskites are highly defect tolerant as bonding and anti-bonding orbitals mostly reside within the bands. Nevertheless, intrinsic defects such as interstitial halide, halide vacancy, and lead vacancy can accompany the formation of either deep or shallow trap states,^80,81^ which are responsible for the material degradation and deterioration of structural stability due to ionic nature of halide perovskites.^82^ Recent studies have shown influence in point defect densities while the perovskites experience strains. Xue *et al.*^46^ used DFT to calculate the strain-dependent formation energies of halide vacancies and found that, compared with the strain-free perovskites, the formation energy of halide vacancies increases upon compressive strain, whereas it decreases under tensile strain, thus resulting in a decrease in non-radiative recombination, as well as a further increase in device performance and lifetime. Ghosh *et al.*^83^ calculated the thermodynamic transition levels for intrinsic vacancy defects of both FAPbI_3_ and FA_0.75_Cs_0.25_PbI_3_ under pressure-induced external stress. They observed a significant shift of iodide vacancies from shallow to deep states in the perovskite materials while exposing them to high pressure >2 GPa, possibly acting as non-radiative recombination centers. However, the transition state levels of vacancy defects show un-noticeable change at low pressures (≤0.5 GPa) and have negligible effects on charge carrier lifetime.

**Fig. 12:**
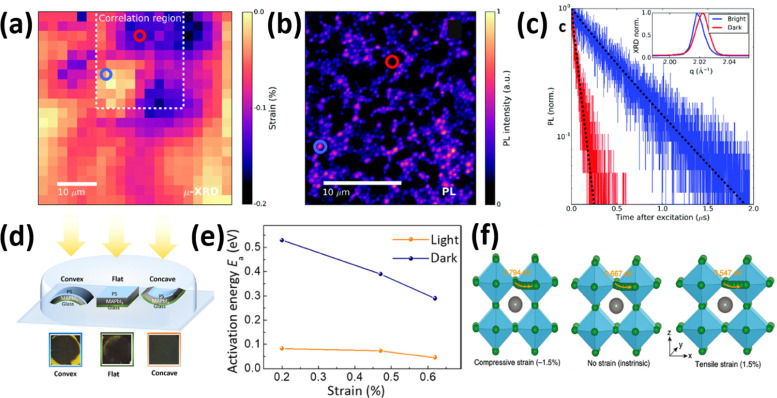
(a) Spatial map of the compressive strained MAPbI_3_ film. The dashed region is the correlation region between μ-XRD and PL. (b) Confocal PL intensity map of the dash region in (a). (c) TRPL decays of the perovskite films corresponding to the highlighted blue and red regions in d. Reprinted with permission,^16^ Copyright 2019, Royal Society of Chemistry. (d) illustration of an experimental setup, consisting of the samples with MAPbI_3_ films deposited on bendable substrates upon different strains, and photographs of the films illuminated under white light with intensity of ∼50 mW cm^−2^ after 500 hours. (e) Activation energy of ion migration changes with strain under dark or illumination. Reprinted with permission.^43^ Copyright 2017, The American Association for the Advancement of Science. (f) Calculated activation energies for halide ion migration in perovskites under compressive strain (−1.5%), no strain (intrinsic) and tensile strain (1.5%). Reprinted with permission,^46^ Copyright 2020, Springer Nature.

Strain-induced structural defects have been reported to show a strong influence on the non-radiative recombination in poly-crystalline perovskite films, which hinders the open circuit voltage of the corresponding solar cell devices. Jones *et al.* demonstrated a direct relation between lattice strain and the increment of defect concentrations and non-radiative recombination sites by utilizing confocal TRPL combined with correlative synchrotron scanning micro-XRD (μXRD) measurements on the same scan area.^16^ After determining the local compressive strain of the film from μ-XRD, they probed the spatial map of that region (Fig. 12a), a confocal PL intensity map (Fig. 12b) of the correlation region highlighted in the strain map in Fig. 12a, and the local time-resolved PL measurements of the corresponding bright and dark regions (red and blue circles in Fig. 12b), as shown in Fig. 12c, representing the recombination charge carriers. Combined with the 〈220〉 μXRD peaks in the inset of Fig. 12c, they found that the dark region has an inferior emission intensity and carrier lifetime, which corresponds to a region with compressive-strained 〈220〉 lattice planes, whereas the bright region possesses a stronger emission and longer carrier lifetime, indicating a comparatively unstrained region. Furthermore, by using a first-principles atomic model and converting the strain map to a relative defect density map, they found a strong anti-correlation between the charge carrier lifetime and defect concentration ratio of a strained crystal. These results suggest that the observed local PL heterogeneity is mainly attributed to the local strain heterogeneity of the perovskite crystal originating from the structural defects such as halide vacancies, which accordingly results in a dramatic non-radiative recombination loss.

### 4.4 Effect on stability

It was reported that tensile strain in perovskite films can reach a value high enough to deform copper (excess 50 MPa in magnitude), which can provide a dramatic driving force to the formation of vacancies, facilitate phase segregation,^84^ accelerate ion migration and subsequent phase transition, and eventually decompose the perovskite films while exposing to humidity, heat and illumination.^45^ However, conversely, researchers observed an increase in stability of perovskite films upon compressive strain. To investigate strain effects on the stability of the perovskite films under illumination, MAPbI_3_ based perovskite films under different bending conditions were sealed in a petri dish and illuminated under white light, as illustrated in Fig. 12d.^43^ After 500 hours illumination under increased lattice strain, a large area of the perovskite film had turned into yellow PbI_2_ as confirmed by XRD. In sharp contrast, the perovskite film exposed to reduced lattice strain remained mostly black with no decomposition under the same condition. It is worth noticing that the strain has been proved to be uniform over the films and the observed non-uniform degradation on the perovskite films was attributed to other factors induced by spin-coating process. In addition, they found that regardless of different substrates, the presence of strain induces accelerated degradation of the perovskite film, however, recovers once the strain has been released. The mechanism of such accelerated degradation was attributed to the increment in ion migration by calculating the activation energies for ion migration of the perovskite films while experiencing enlarged strain (from convex, flat to concave), based on the Nernst–Einstein relationship:6
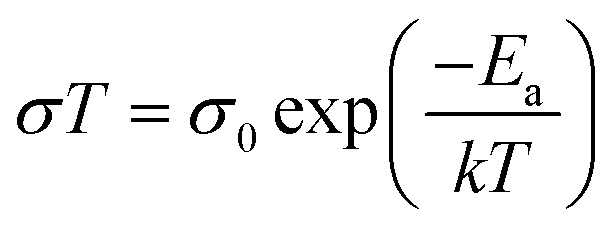
where *σ* is conductivity, *T* is temperature, *σ*_0_ is conductivity constant in the absence of ionic motion, *k* is Boltzmann constant and *E*_a_ is the activation energy for ion migration. As shown in Fig. 12e, it was observed that the activation energies for ion migration in the perovskite film decrease when the strain is enlarged, which also significantly decreases under illumination. These results suggest that both enlarged strain and illumination can induce accelerated ion migration, leading to a faster degradation in the perovskite films.

Complementary to this experimental evidence, calculations have also been carried out to determine activation energies (*E*_a_) for the vacancy-assisted migration of halide ions in perovskite films while experiencing strains.^46^*E*_a_ in strain-free CsPbI_2_Br perovskite film was calculated as 0.667 eV, which increases to 0.794 eV under compressive strain and decreases to 0.547 eV under tensile strain (Fig. 12f). These results confirm the accelerated ion migration in perovskite films under enlarged tensile strain, leading to a deterioration of perovskite stability. Meanwhile, it was also suggested that compressive strain can decelerate halide ion migration in perovskites, thus expecting to improve the intrinsic stability of the material.

Moreover, Rolston and co-workers examined the stability of MAPbI_3_ films after external compressive or tensile strain by varying the applied pressure from −130 MPa (compressive strain) to 130 MPa (tensile strain) on the perovskite films.^45^ Then they exposed the stressed films to humid air at room temperature (85% R.H., 25 °C) and dry air with elevated temperature (25% R.H., 85 °C), respectively. Photographs were recorded after 24 h aging. It can be clearly seen that, in both conditions, the perovskite films under tensile strain displays a pronounced visible degradation from black phase MAPbI_3_ into yellow color PbI_2_, whereas, the compressed perovskite films mostly remained in the pristine black perovskite phase.

## 5. Strategies to improve device performance and stability of PSCs

**Fig. 13:**
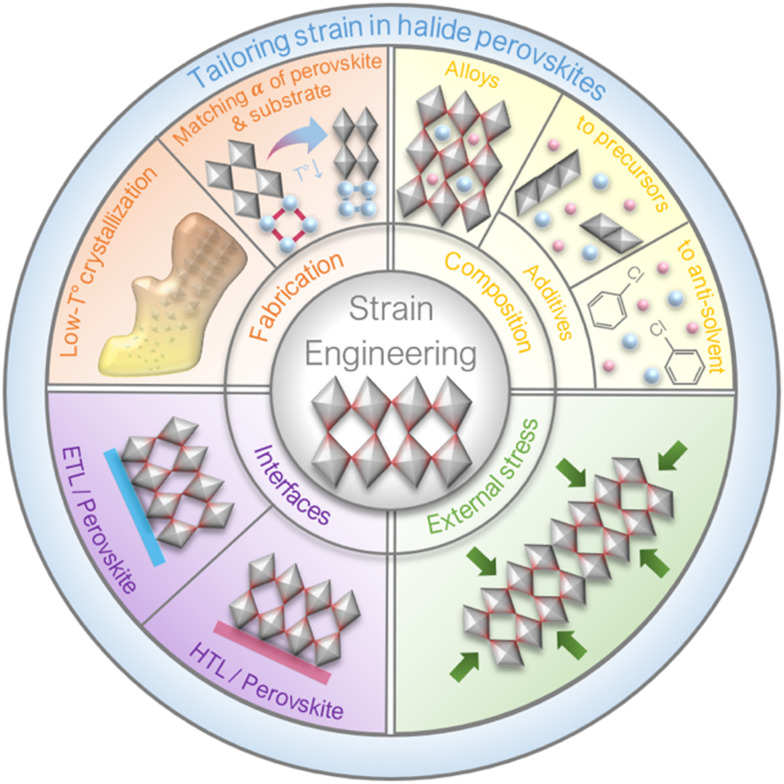
Overview of strain engineering strategies on improving device performance and stability.

As discussed above, strain plays an important role in affecting the bandgap, charge carrier transport, defect properties and non-radiative recombination, as well as the stability of the perovskite films. Therefore, strain can induce both positive or negative effects on the performance and stability of PSCs, various strategies to adjust strain effect will be discussed in this section, as summarized in Fig. 13.

### 5.1 Engineering of the fabrication process

It is well-known that thermal expansion mismatch between the perovskite film and the substrate during thermal annealing process is one of the major causes of strain in perovskite fabrications. Thus, (1) lowering the formation temperature of the perovskite film or (2) reducing the thermal coefficient mismatch between the perovskite layer and the substrate are the most straightforward ways to reduce the tensile strain stemming from the thermal expansion mismatch during the fabrication process.

Zhao *et al.* proved that the formation temperature of a perovskite film is a critical factor that determines the amount of its lattice strain.^43^ Instead of annealing at high temperature, they fabricated the MAPbI_3_ perovskite film under room temperature by evacuating the film for 3 days and found the formation of a strain-free perovskite film. In comparison, the perovskite films formed through annealing under different temperatures exhibited lattice strain (similar in value regardless of the applied temperature). It is worth to mention that, the already formed perovskite films are insensitive to post-annealing, as they found that the strain-free/strained perovskite film remain unstrained/strained after being heated at 100 °C for 4 h (20 h). This is due to the strong adhesion between the perovskite layer and its contact layer once the perovskite film was formed, which is also consistent with other reports in literature.^46,85^

The most commonly used anti-solvent engineering strategies require annealing to evaporate the residual solvent such as chlorobenzene in order to form a highly crystalline perovskite film, which consequently causes tensile strain while cooling down to room temperature. Alternatively, Rolston *et al.* proposed a bath conversion method, which enabled the formation of a less-stressed perovskite film at room temperature.^45^ The bath-converted perovskite film formed directly under room temperature by submerging the spin-coated perovskite film in a very low boiling temperature solvent, diethyl ether, possessed a largely reduced residual strain, which would not be influenced by any further procedures requiring elevated temperatures.^45^ However, although lowering the annealing temperature for perovskite formation can dramatically diminish the tensile strain in the film, it is worth noticing that this strategy might, on the other hand, result in a decrease in device performance, mainly due to the low quality of the perovskite films formed under low temperature.

Alternatively, using a substrate with higher thermal expansion coefficient, such as polycarbonate (PC) or polyethylene terephthalate (PET), to replace the commonly used indium tin oxide (ITO) or fluorine-doped tin oxide (FTO) glass substrates is also a promising strategy to reduce the mismatch between the perovskite and the substrate,^46^ since the thermal expansion coefficient of perovskites with various compositions are rather similar.^86,87^ Nevertheless, the device performance based on those flexible substrates is still not comparable with those on rigid substrates, which is mainly attributed to their resistivity and transmittance properties, as well as the effect of the perovskite films formed on top.

### 5.2 Compositional engineering on the perovskite layer

As mentioned above, intrinsic strain normally occurs during the formation of a perovskite film, compositional engineering of the perovskite precursor solution is therefore considered as an effective strategy to modulate the strain of a perovskite film.

#### 5.2.1 Alloying in perovskite composition

Different A-site cations can dramatically influence the intrinsic strain behavior of an as-prepared perovskite film, based on their difference in molecular properties.^88^ Local lattice strain of single-cation halide perovskite, such as FAPbI_3_, induced by ionic size mismatch between A-site cation and the lead halide cage can lead to a lattice distortion and [PbI_6_]^4−^ octahedra tilting. Saidaminov *et al.*^89^ reported that such strain can be relaxed *via* point defect formation with oxygen and water molecules, which however, would facilitate the perovskite decomposition. Otherwise, incorporating small ions such as Cs^+^/MA^+^ into FAPbI_3_ can effectively relax the lattice strain of FAPbI_3_ and prevent the formation of vacancy defects, leading to a dramatic improvement in device performance and long-term stability, which is also in agreement with other reports.^66,90,91^

It is worth noticing that A-site alloying can indeed improve the stability of halide lead perovskites by releasing the strain through lattice shrinkage, however, the spacing of [BX_6_]^4−^ framework is the main factor that determines the lattice constant. Therefore, regulation of the remaining lattice strain on B/X sites of the perovskite is also desirable to understand. Incorporating possible isovalent dopants in B/X sites of the CsMAFA-based perovskites has been investigated. Among various candidates, PSCs incorporating with Cl^−^ and Cd^2+^ show similar PCEs with the control devices, however a significant enhancement in stability under various conditions, especially the devices incorporated with Cd^2+^.^89^ Besides, Shai *et al.* reported that introducing Zn^2+^ into MAPbI_3−*x*_Cl_*x*_ perovskite (with Zn : Pb ratio less than 1 : 100) can release the lattice strain during an appropriate lattice constriction within B–X framework, leading to reduced crystal defects and enhanced intrinsic stability.^92^ Also, the incorporation of pseudo-halide into perovskites was reported to induce the relaxation of lattice strain. Zhang *et al.* used NH_4_BF_4_ and occupation of BF_4_^−^ in the X site of perovskite (FAPbI_3_)_0.83_(MAPbBr_3_)_0.17_ resulted in a lattice expansion verified by a peak shift of (001) diffraction toward lower 2*θ*.^93^ The authors suggested that the lattice relaxation could be achieved by a weakened bond of Pb–BF_4_ possibly due to their weaker hybridization than that of Pb–I. The authors empirically showed the effective suppression of defect formation and reduced density of defects which were ascribed to the strain relaxation. Similarly, Oh *et al.* suggested that the lattice strain relaxation is realized by the intercalation of pseudo-halide (BF_4_^−^) into mixed Pb–Sn perovskite (FA_0.5_MA_0.5_Pb_0.5_Sn_0.5_I_3_) that is more vulnerable to lattice strain because of the different ionic radii between Sn (1.18 Å) and Pb (1.19 Å) than pure lead halide perovskites.^94^ The authors proposed that strain relaxation increases the formation energy of iodine vacancy and inhibit the formation of metallic species Pb^0^ and Sn^0^, which would degrade device performance.

#### 5.2.2 Additive in perovskite composition

**Fig. 14:**
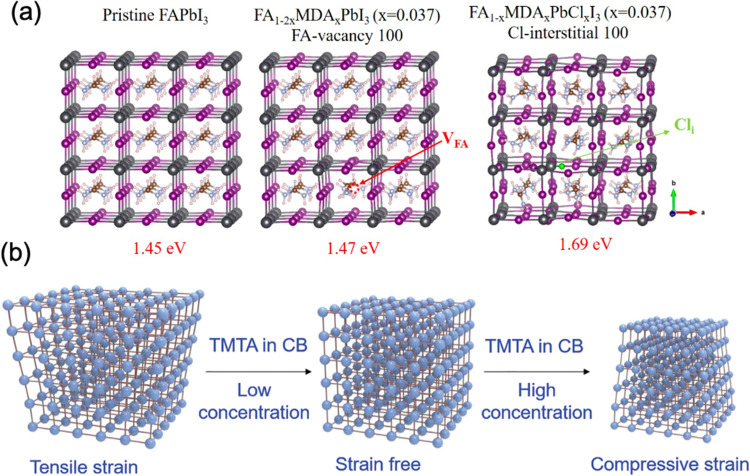
(a) Structures of pristine FAPbI_3_, FA_0.926_(V_FA_)_0.037_MDA_0.037_PbI_3_ and FA_0.963_MDA_0.037_PbI_3_(Cl_i_)_0.037_. Reprinted with permission.^96^ Copyright 2019, The American Association for the Advancement of Science. (b) Schematic illustration of strain regulation of perovskite with different concentrations of TMTA in CB. Reprinted with permission^99^ Copyright 2021, Wiley-VCH.

Additives in perovskite compositions without incorporating into the perovskite lattice have been developed to tailor the strain of a perovskite film. Wang *et al.* doped aluminium acetylacetonate (Al-acac_3_) into the perovskite precursor solution of MAPbI_3_, and found that the presence of a small amount of Al^3+^ (<0.3 mol%) can enhance the crystallinity of the perovskite film with reduced microstrain, thus leading to an improvement in the device performance.^95^ Min *et al.* found a more stable structure of α-phase FAPbI_3_ by introducing methylenediammonium choride (MDACl_2_) into the perovskite precursor, owing to the lattice strain relaxation of partial substitution of I site by smaller Cl^−^ ions, as illustrated in Fig. 14a.^96^ The introduction of 3.8 mol% MDACl_2_ can suppress the defect formation and increase the charge carrier lifetime of the perovskite film, resulting in excellent device performance with a certified PCE of 23.7%. Kong *et al.* employed 2-diethylaminoethylchloride hydrochloride (DEAECCl) into the perovskite solution.^97^ The internal tensile strain of 3D perovskite film has been efficiently relieved after the introduction of residual flexible 1D perovskitoid. As a result, the target devices retained over 90% of their initial performance after more than 2000 h ageing conditions. Similarly, Wang *et al.* introduced n-butylammonium cation (BA^+^) into a mixed-cation (FA^+^/Cs^+^) and mixed-halide (I^−^/Br^−^) perovskite and observed a reduced lattice constant with the increment of the BA^+^ content, which was speculated as the released strain. As a result, the partial substitution of FA^+^/Cs^+^ by BA^+^ enhanced both device performance and stability.^98^

Very recently, Doherty *et al.* found that the stabilized FA-rich perovskites with A-site cation (such as Cs, MA cations, or their combination) alloying are still noncubic and exhibit small (∼2°) octahedral tilting at room temperature, which can only be observed by local nanostructure characterization techniques such as SED (which was described in Section 3.2).^31^ In this case, although the perovskite bulk is phase-stable macroscopically, the heterogeneous cation distributions could still form microscopically unstable regions, which would eventually create local trap-assisted performance losses and degrade the device. Moreover, halide mixing was found to induce additional influence in the observed degree of octahedral tilting. As an alternative, the authors demonstrated a strategy to effectively stabilize the α-FAPbI_3_ by templating the perovskite growth and its octahedral tilting in order to conjugate the cation-alloying approaches, homogenize nanoscale phase stability and eliminate residual traps. They found that octahedral surface functionalization with an ethylenediaminetetraacetic acid (EDTA) could induce a slight octahedral distortion across the perovskite film, however to a degree that inhibits the transition from corner-sharing to face-sharing structures without compromising its optoelectronic properties. As a result, the templated FAPbI_3_ film exhibits outstanding stability upon external stressors (thermal, environmental and light).

#### 5.2.3 Additive in antisolvent

Additive in antisolvent has been investigated to regulate the top perovskite region, where the most serious tensile strain occurs.^88^ For example, Zhang *et al.* demonstrated an *in situ* crosslinking-enabled strain-regulating crystallization (CSRC) method by introducing trimethylolpropane triacrylate (TMTA) into the antisolvent of chlorobenzene (CB) to confine the thermal expansion.^99^ Remarkably, they found that the concentration of TMTA in CB can precisely regulate the lattice strain of the perovskite film during annealing process, as presented in Fig. 14b. As a result, the CSRC approach improved a PSC in terms of PCE and long-term stability under storage, thermal and light conditions.

### 5.3 Interfacial engineering

Compensation of intrinsic tensile strain in the perovskite film by adjusting its adjacent layers with lower or higher *α* has been proved as a promising and effective approach for strain management in PSCs. Although using a substrate with higher *α* or decreasing the annealing temperature of the perovskite layer can indeed reduce the *α* mismatch between the perovskite and the substrate as discussed in Section 5.1, these strategies normally result in either low quality perovskite films or unsatisfied interconnection with inferior carrier collection, which is therefore unfavorable for the potential device efficiency and long-term stability.^66^ Thus, it is important to control the residual strain of the perovskite but concurrently tuning the strain at the interfaces of ETL/perovskite and perovskite/HTL. Several studies have shown encouraging enhancement in device performance and stability utilizing strain-compensate strategy by applying suitable functional layers at the interfaces of the perovskites.

#### 5.3.1 ETL/perovskite interface

**Fig. 15:**
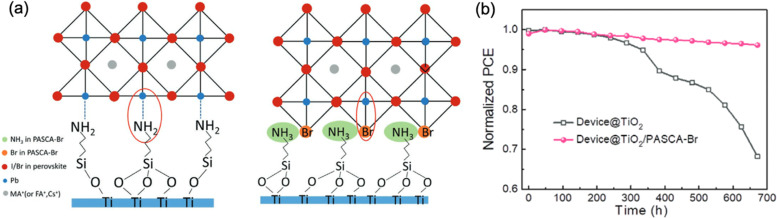
(a) Lattice structure of APTES and PASCA-Br modified interfaces. (b) Stability of the devices with and without PASCA-Br modification after ageing for 1 month. Reprinted with permission^100^ Copyright 2020, Wiley-VCH.

Zhang *et al.* adopted a protonated amine silane coupling agent (OC_2_H_5_)_3_–Si–(CH_2_)_3_–NH_3_Br (PASCA-Br) as an interlayer between TiO_2_ and perovskite, which can not only anchor to the TiO_2_ layer through Si terminals but also supply as structure component in mutilated octahedra of the perovskite unit through the R–NH_3_Br terminals, in comparison with the traditional (OC_2_H_5_)_3_–Si–(CH_2_)_3_–NH_2_) (APTES), leading to a reduced lattice distortion (Fig. 15a).^100^ Remarkably, the stretchable R–NH_3_Br growth sites help to release the perovskite lattice stress, thus leading to inhibited interfacial strains. As a result, the target devices delivered PCEs of 21.6% on glass substrate and outstanding long-term stability (Fig. 15b). Guanidinium bromine (GABr) has been applied by Zhu's group to assist a secondary growth at the surface of the perovskite film, which reduced the film microstrain and suppressed non-radiative recombination, leading to a significant increase in device *V*_OC_.^101^

In addition, passivating 3D perovskite by forming an additional 2D layer on top is a widely used strategy to enhance surface hydrophobicity, reduce surface recombination and defect states toward more efficient and stable PSCs. However, Zhang *et al.* found instability of such 2D/3D heterostructure under regular thermal processing conditions, due to the lattice expansion of the strained 2D PEA_2_PbI_4_ perovskite layer.^85^ Therefore, they proposed a strain-compensation strategy by introducing PCBM with a low thermal expansion coefficient than that of PEA_2_PbI_4_, as an external compressive strain layer to counteract the lattice expansion. Meanwhile, [PbI_6_]^4−^ octahedra in PEA_2_PbI_4_ embedded between the 3D perovskite and PCBM layer can suppress the ion migration due to the strong interaction with both layers. HRTEM revealed that the diffusion of small ions caused by thermal annealing forms 2D passivating structures along grain boundaries, thus reducing the recombination velocity. Due to the combinative effect of diffusion passivation and stress compensation, they have boosted *V*_OC_ to 1.1 V, along with a *J*_SC_ of 22.76 mA cm^−2^ and FF of 79.3%, resulted in a PCE of 21.31% measured in the laboratory and an independently certified PCE of 20.22%.

#### 5.3.2 Perovskite/HTL interface

**Fig. 16:**
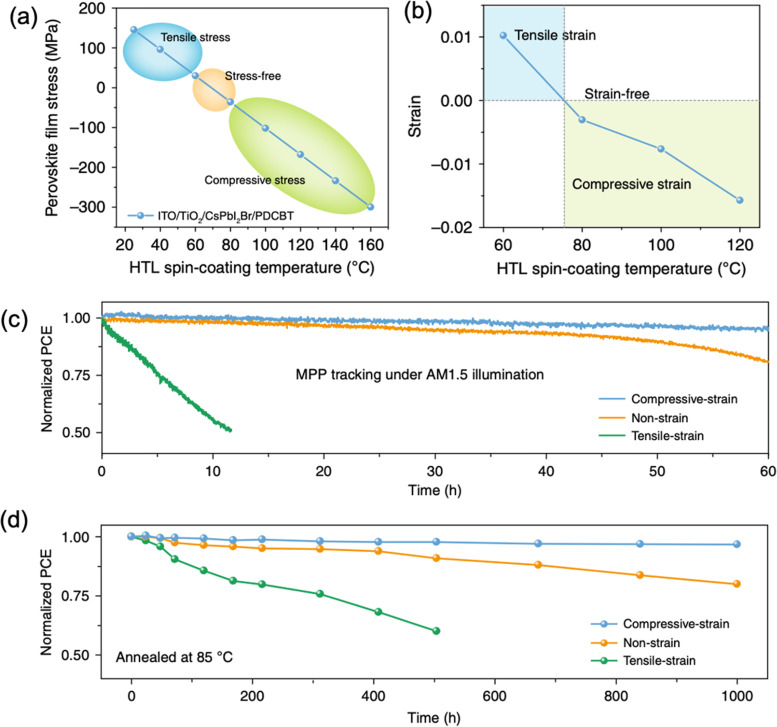
(a) Calculated and (b) measured CsPbI_2_Br perovskite film stress variation under different spin-coating temperature of PDCBT. Stability of the devices with compressive-strain, non-strain and tensile-strain under (c) MPP tracking with AM1.5G illumination and (d) annealing temperature at 85 °C. Reprinted with permission,^46^ Copyright 2020, Springer Nature.

Xue *et al.* utilized a HTL, poly[5,5-bis(2-butyloctyl)-(2,2-bithiophene)-4,4′-dicarboxylate-*alt*-5,5′-2,2′-bithiophene] (PDCBT), with rich carbonyl anchoring groups to build a strong perovskite (CsPbI_2_Br):HTL interface for strain transfer and found a linear correlation between stress and annealing temperature of the HTL as shown in Fig. 16a, where the increased annealing temperature of HTL offsets the residual tensile strain of the perovskite.^46^ An eliminated tensile strain is observed in the perovskite film when the PDCBT layer was spin-coated at around 80 °C, which becomes compressive strain as the spin-coating temperature increases (Fig. 16b). Remarkably, both devices with strain-free and compressive-strained perovskite films show outstanding stability under both MPP tracking at continuous one sun illumination (Fig. 16c) and elevated temperature (85 °C) conditions (Fig. 16d). These results indicate that the residual tensile strain in the perovskite film can be modulated by depositing the top hole transporting layer at higher temperatures to introduce a more stable strain-free or compressive-strained perovskite films. In addition, Cui's group employed a polymerizable propargylammonium (PA^+^) at the 3D perovskite surface and grain boundaries to form cross-linked 1D/3D perovskite heterostructure, which changes the residual 3D perovskite film from tensile strain to compressive strain and thus benefits the corresponding device performance and long-term stability.^102^

Despite of only considering interfacial strain relaxation with functional materials, our group recently realized that the combined solvent could also play an important role in releasing the residual strain of a perovskite film.^103^ We found that comparing with isopropanol (IPA), employing cyclohexylmethylammonium iodide (CMAI) in chloroform (CF) for post-treatment on a perovskite film, can create a strain-free surface owing to the slow ion exchange between the CMA^+^ and FA^+^, leading to an enhanced long-term stability of the PSCs.

### 5.4 External strain

**Fig. 17:**
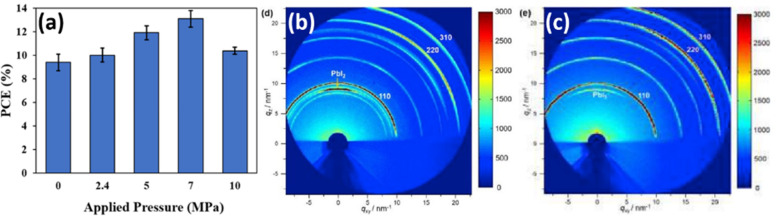
(a) Change in PCE as a result of applied pressure. Reprinted with permission.^104^ Copyright 2021, Wiley-VCH. GIXRD patterns of (b) reference sample and (c) sample manufactured *via* PASP method. Reprinted with permission.^105^ Copyright 2020, Elsevier.

Oyelade *et al.* have demonstrated the possibility to enhance the performance of the PSCs by pressure-assisted fabrication technique.^104^ The PCE of manufactured devices improved from 9.84% to 13.67%, by applying additional pressure in the range of 0–7 MPa (Fig. 17a). The authors attributed growing initial trends in the PCE (for pressures below 7 MPa) to a better interfacial contact and densification of the mesoporous layer. However, for the pressure values beyond 7 MPa, the reduction of PCE was affiliated with perovskite layer fragmentation.^87^ While the mentioned mechanisms can partially explain the PCE change, it is clear that applied pressure also causes strain in perovskite lattice, which affects the opto-electronic properties of perovskite films and device performance. Luo *et al.* also demonstrated pressure-assisted solution processing (PASP) method to control the perovskite nucleation and growth for controllable fabrication of highly crystallized perovskite films with micron-sized grains and microsecond-range carrier lifetimes.^105^ It is expected that such crystallization route also results in specific grain orientation spread and modulation of strain, as we have shown earlier in Section 2.2.2. This crystallization approach also resulted in higher tolerance of perovskite films to moisture-induced degradation and to ion migration. Consequently, PSCs made *via* PASP method had a champion PCE of 20.74% under applied pressure of 4.9 kPa and extraordinary stability against humidity and continual light illumination. Notably, from GIXRD measurements in Fig. 17b and c, the pressure-assisted films exhibit Debye–Scherrer rings more homogeneous in intensity, meaning that scattering is less influenced by incident angle, which is usually a signature of a better lattice isotropy. Therefore, the 2D XRD data of the PASP-sample (Fig. 17c) looks more like 1D, unlike the control perovskite film (Fig. 17b).^105^

The continuous light soaking by standard one-sun (100 mW cm^−2^) has also been reported to cause a large and uniform lattice expansion of hybrid perovskite films as shown in Fig. 6c.^56^ Evidence shown in the *in situ* GIWAXS measurements on the FA_0.7_MA_0.25_Cs_0.05_PbI_3_ perovskite film with increasing illumination time from 0 to 180 min. No phase segregation or degradation emerged under light illumination, however, a uniform shift toward lower scattering vector *q* values is clearly observed in all diffraction peaks, which corresponds to an isotropic increase in lattice constant *d* (lattice expansion), as plotted in Fig. 6b. As a result, they found that the relaxation of local lattice strain upon light soaking can not only enhance the crystallinity of the perovskite film, but also lead to a lower energetic barrier at the interface between perovskite and the adjacent contact layer to guarantee an efficient charge collection and suppress the non-radiative recombination in the bulk. Consequently, it boosts the solar cell performance without detrimental effect to its stability.

## 6. Summary and perspective

### 6.1 Summary

Strain in halide perovskites and perovskite solar cells is increasingly gaining attention of the research community. To be able to control its impact on the optoelectronic properties and device stability, a deeper understanding of the concept and its effect on halide perovskites is necessary and important to investigate. Herein, we presented a systematic overview of the definition, origin, various characterization methods for probing the strain, implications on perovskite films, as well as regulation strategies for strain engineering in improving PSCs device performance and long-term stability.

Strain in perovskites is normally generated by:

(1) internal stress induced by the non-periodicity of crystal lattice;

(2) external stress caused by lattice and thermal expansion mismatch or external stress sources such as light, temperature, pressure or applied bias.

Such strain can be detected by various of characterization methods, on both macro-scale techniques with high spatial averaging, such as XRD/GIXRD, Raman spectroscopy, PFM, TEM, GIWAXS, PL, TRPL, TPC, and also micro-/nano-scale techniques, such as SED and nano-XRD and CDI, which allow highly-resolved analysis of strain.

Various implications of strain on perovskite films have also been summarized, in terms of its effect on bandgap, charge carrier transport, defect properties and non-radiative recombination, as well as crystal film stability. From the discussed literature reports, we can recognize the following trends:

(1) A dramatic redshift with accordingly narrower bandgap upon compressive stress, however, a blueshift with accordingly wider bandgap upon tensile strain on a perovskite material. Exceptions such as bandgap of 1D MAPbI_3_ exhibit near parabolic response upon strain, which increases under both compressive and tensile strain.

(2) A more upward lifting of VBM than CBM under compressive strain, leading to a higher carrier mobility and a better energy alignment between the VBM of the perovskite and HTL. In contrast, a VB downward bending under tensile strain would induce an unfavorable energy level gradient for hole extraction and diffusion, causing deeper level defects in perovskites.

(3) A decreased bulk conductivity of perovskite under tensile strain, however, an increase under compressive strain based on DFT calculations.

(4) An increase in formation energy of halide vacancies upon compressive strain, and a decreasing formation energy under tensile strain, the latter leading to an increase in non-radiative recombination of a perovskite film.

(5) A more stable perovskite film after compressive stress, however, an accelerated degradation perovskite film after tensile stress.

A range of strategies to control strain have been explored in literature. Based on this review, we can consolidate the following effective strategies to improve device performance and stability of PSC:

(1) Reducing detrimental lattice strain during crystal formation *via* engineering of the fabrication process by lowering the formation temperature of the perovskite layer or reducing the thermal coefficient mismatch between the perovskite layer and the substrate.

(2) Compositional engineering on the perovskite layer through cation alloying, additive in perovskite composition or antisolvent.

(3) Interfacial engineering at the interfaces between the perovskite layer and its adjacent layers through functional groups or additional interlayers.

(4) Engineering of external strain, with sources such as pressure and light. However, these effects are not fully understood and require further investigation.

### 6.2 Perspective

Unprecedented success has been achieved and great efforts have been made in the field of PSCs to push the PCE over 25%, which corresponds to ∼80% of its (theoretical) Shockley–Queisser (SQ) limit.^1^ Recent progress is mainly attributed to the development of key strategies that effectively reduce the defects on the surface of the perovskite layer and minimize non-radiative recombination at the interfaces, thereby enhancing device efficiency. This review highlights strain management in PSCs as one of the promising routes to manufacture devices with PCEs exceeding 26% and closer to the radiative limits.

Since PCE enhancement effectively translates into an increase in *J*_SC_, FF and *V*_OC_, one could consider the ratio of these properties to their SQ limit value for a solar cell with a specific bandgap as a figure of merit describing the remaining room for improvement.^106^ It has been noticed that most of the record-holding PSCs shown in the past demonstrated comparable *J*_SC_/*J*^SQ^_SC_ ratios of ∼0.9 and is more likely to be related to the optical losses (particularly parasitic absorption in the substrate), rather than the perovskite absorber layer. On the other hand, the improvement of the product of *V*_OC_ and FF compared to their SQ limits ([*V*_OC_ × FF]/[*V*^SQ^_OC_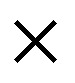 FF^SQ^]) is directly linked to the increase in PCE/PCE^SQ^ ratio. Therefore, improvement in PSC performance is likely to be achieved *via* the elimination of bulk- and surface-related defects, improvements in carrier collection/transport, and enhanced radiative efficiencies, which are all closely tied to *V*_OC_ and FF. Thus, we expect that carefully tailoring of strain in halide perovskites can lead to fabrication of devices with nearly ideal interfaces, effectively reducing the ([*V*_OC_ × FF]/[*V*^SQ^_OC_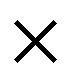 FF^SQ^]) gap.

We conclude that the majority of literature reports demonstrated that tensile strain deteriorates the performance of a PSC and accelerates its degradation. In contrast, moderate compressive strain can improve the optoelectronic properties and stability of the perovskite films as well as the corresponding solar cells. Therefore, the aim of strain regulation in PSC should be to release its residual tensile strain and introduce controlled compressive strain to the perovskite bulk crystal layer or interfaces. Based on the knowledge collected in this literature review, we propose the following pathways for future strain engineering in PSC:

(1) Investigate novel or modified synthetic routes toward high quality perovskite films, especially under low processing temperatures.

(2) Explore new additives or interfacial materials between the perovskite and charge extraction layers to adjust/compromise the strain in perovskites without introducing phase and structural heterogeneity, even on nanoscale.

(3) Develop novel substrates with similar thermal coefficients as perovskites, having comparable optoelectronic properties with traditional rigid substrates, and ensuring the formation of high-quality perovskite film deposited on top of it.

Considering that the importance of strain for the PSC is increasingly moving in the focus, a further and deeper understanding of its origin, impacts, and means for control are essential to pursue highly efficient and more stable and push perovskite PV closer to commercialization.

## Conflicts of interest

There are no conflicts to declare.

## Supplementary Material
